# Mathematical modeling for the prediction of some quality parameters of white rice based on the strength properties of samples using response surface methodology (RSM)

**DOI:** 10.1002/fsn3.1703

**Published:** 2020-06-14

**Authors:** Nasrollah Fazeli Burestan, Amir Hossein Afkari Sayyah, Ebrahim Taghinezhad

**Affiliations:** ^1^ College of Agriculture and Natural Resources University of Mohaghegh Ardabili Ardabil Iran; ^2^ Moghan College of Agriculture and Natural Resources University of Mohaghegh Ardabili Ardabil Iran

**Keywords:** quality properties, response surface methodology, single kernel, strength properties, white rice

## Abstract

One of the major problems in predicting the quality properties of rice is that conducting experiments in the food industry can be highly expensive. The objective of this study was to predict some quality properties in varieties (*Domsiah, Hashemi, Dorfak*, and *Kadus*) via compression test at moisture levels 9 and 14% w.b. Based on historical data design, RSM was used to model and estimate of dependent variables (amylose (AC) and protein content (PC), gelatinization temperature, gel consistency GC), minimum (Min.V), final (FV), breakdown (BDV) and setback viscosity (SBV), peak time (PT) and pasting temperature (Pa.T)) through independent variables (the rate of force, deformation, rupture energy, tangent, and secant modulus). An ANOVA test showed that models were significant (*p* < 0.05). The most appropriate model for response variables prediction of AC and GC (*Kadus* 14%), PC (*Domsiah* 9%), Min.V, FV, and SBV (*Dorfak* 9%), BDV (*Dorfak* 14%), PT (*Hashemi* 14%), and Pa.T (*Kadus* 9%) was $R_{\text{pred}}^{2}$ as 0.86, 0.85, 0.93, 0.955, 0.953, 0.94, 0.94, 0.86, and 0.91, respectively, with the most appropriate optimal values as 23.52%, 48, 10%, 164.95 RVU, 304.12 RVU, 162.66 RVU, 64.52 RVU, 6.09 min, and 92.45°C and desirability as 0.91, 0.95, 0.95, 0.80, 0.89, 0.83, 0.84, 0.89, and 0.96, respectively. The optimal values of the independent variables have a decreasing trend, and the optimal values of the response variables are proportional to the optimal conditions. The results indicated that the RSM could be quite useful in the optimization of the models developed for predicting the rice quality properties.

## Graphical abstract

The development of more inexpensive methods can help precipitate the marketing process of the rice. The results are expected to lead to the development of mechanical tests to prediction of the quality. The results can be used for designing and developing devices to test the quality indices of kernels; similar to those are being used to test the wheat quality according to single kernel characterization (SKCS).

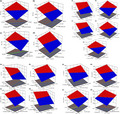

**Practical Applications**

All existing methods for measuring the rice quality indexes are labor‐expensive or time‐consuming. The development of more inexpensive methods can help precipitate the marketing process of the rice. The results are expected to lead to the development of mechanical tests to prediction the quality. The results can be used for designing and developing devices to test the quality indices of kernels, similar to those are being used to test the wheat quality according to single kernel characterization (SKCS).

## 1 INTRODUCTION

Rice is one of the most dominant food crops in the world. The commercial value of rice is largely determined by the quality characteristics of milled rice (Vithu, Tech, & Moses, 2016). Eating and cooking of rice considerably affect its acceptability among consumers. Quality indicators such as amylose content, gelatinization temperature, gel consistency, and pasting properties are essential in the final quality of rice (Ferreira, Oliveira, Pathania, Almeida, & Brites, 2017). To determine some strength properties of rice grain used a single kernel to estimate rice quality (Yu et al., 2019). With all its simplicity, the measurement of strength properties is very important in determining the quality of rice, which has been studied by various researchers in different grains, especially in cereals. Although the quality properties are determined using quality analysis tools in food industry laboratories, experiments with these tools are highly protracted and expensive. Thus, the development of more inexpensive and convenient methods can help precipitate the marketing process of the product. Up until now, several studies have been conducted on the quality and strength properties of rice. However, very few of them have addressed the relationship between these properties.

The mechanical attributes of individual kernels are more relevant to the quality properties of the various cereals. The relationship between kernel hardness, a quality parameter used for grading cereals, e.g., wheat, and some mechanical attributes, e.g., the maximum compressive force (Osborne & Anderssen, 2003). They are also used in the determination of the relationship between hardness and quality parameters to grade cereals. In their study, Cao, Nishiyama, and Koide (2004) explored the effect of moisture content (MC) on some mechanical properties of brown rice. They showed that MC drastically affects the mechanical properties; that is, compressive strength and tensile strength decreased with increasing MC. Some researchers have attempted to extract the strength attributes of the rice grain. For example, Kamst, Vasseur, Bonazzi, and Bimbenet (1999) reported that Young's moduli from the diametral compression tests are not significantly different from those from uniaxial compression tests. The repeatability of the diametral compression tests is of the same order as the repeatability of uniaxial compression tests.

RSM consists of a collection of statistical and mathematical techniques that are used to improve, expand, and optimize processes that can demonstrate the relationship between response and independent variables through mathematical models (Montgomery, 2008). Numerous studies have exploited RSM. For instance, Taghinezhad and Brenner (2016) investigated the mathematical modeling of starch gelatinization and some quality attributes of parboiled rice through RSM. They asserted that this model is the most appropriate model for predicting the degree of starch gelatinization, hardness, lightness, color value, and head rice yield of samples (*R* ^2^ ≥ 0.86). Moreover, Yousaf et al. (2017) studied the optimization and mathematical modeling of the quality attributes of parboiled rice using RSM. They posited that it is the most appropriate model that can predict the effect of temperature, soaking time, and steaming time on head rice yield, hardness, cooking time, lightness, and color of samples (*R* ^2^ ≥ 0.88). They also introduced an optimal model for their research.

A review of the literature revealed that some studies have been conducted on optimizing the quality of different types of grainy cereals, especially rice. Besides, they have mainly focused on a very limited number of factors involved. The effect of mechanical properties on the quality properties of rice has either not been addressed or been regarded attentively. Therefore, it is necessary to study the effects of mechanical properties on the quality attributes of rice through analyzing single kernels of rice. In this study, some strength components of rice grains were extracted from a series of compression tests performed on four typical Iranian rice varieties and assessed by RSM due to the importance of strength parameters of rice and the feasibility of SKCS in quality evaluation. The factors focused in this study were strength components including the rate of force, deformation, rupture energy, tangent and secant modulus, and the quality indices of processed rice including amylose content (AC), protein content (PC), gelatinization temperature (GT), gel consistency (GC), minimum viscosity (Min.V), peak viscosity (PV), final viscosity (FV), breakdown viscosity (BD.V), setback viscosity (SB.V), peak time PT), and pasting temperature (Pa.T). With fewer expenses, and less acceptable accuracy via focusing on the mechanical properties of single kernels, the results of this research are expected to lead to the development of instruments for determining the quality of rice. Finally, the independent variables were optimized for the prediction of the quality characteristics (dependent variables) of rice samples.

## 2 MATERIAL AND METHODS

### 2.1 Row specimen provision

The four common varieties of rice bulk samples were acquired in sufficient amounts directly from the field of Rice Research Institute located in Rasht, Iran. The initial moisture content (MC) was determined by placing three samples each weighing 15 g into an oven with a temperature of 130°C for 24 hr (Li, Li, Ding, Chen, & Ding, 2014). The MC of the samples ranged from 15% to 16% (w.b). Then, to reduce MC to 9% and 14%, the paddies were spread on a flour in thin layers and dehydrated by a laboratory drier at the temperature of 35°C. After crushing their skin, they were milled using a UDY cyclone miller (USA) under the same condition and then stored at the temperature of about 4°C for the subsequent experiments.

### 2.2 Compression test

The strength of rice grain was measured using a uniaxial compression test, which was performed on whole and sound grains laying on the floor on their side by loading tow flat plate. The tests were conducted by SANTAM uniaxial compression testing machine (STM‐20, Iran) according to the standard (ASAE, 2009) at the deformation rate of 1.24 mm/min (Arana, 2012). From the force–deformation plotting, values of several parameters were measured such as force value at the deformation of 0.05 mm (F_0.05_), rupture force (F_max_), maximum deformation (D_max_), rupture energy (EF), tangent modulus at the deformation of 0.05 mm (Et_0.05_), maximum tangent modulus (Et_max_), secant modulus at the deformation of 0.05 mm (Es_0.05_), and maximum secant modulus (Es_max_). According to the data obtained from the tests, the first point that underwent force loss as the deformation increased was the rupture point. The amount of force at this point was measured as the F_max_. F_max_ was obtained by measuring the area under the curve of force–deformation to the rupture point (Li et al., 2014). Tangent modulus; the slope of the curve at any point and secant modulus; and the slope of the line from the origin to any point on the force–deformation curve (Henry, Zhang, & Onks, 2000) were measured at different points on the curve.

### 2.3 Determination of the quality attributes of rice

Chemical properties including AC, GT, GC, and PC and the starch physicochemical properties including Min.V, P.V, F.V, BD.V, SB.V, PT, and Pa.T were measured as dependent variables. The AC was determined through colorimetry at the wavelength of 620 nm by forming an iodine–starch complex (Kong, Zhu, Sui, & Bao, 2015). In order to determine the GT, Little's method was used (champagne et al., 1999). Then, six whole grains of the white rice were added to Petri dishes and 10 ml of potassium hydroxide (KOH) 1.7% was added to them. Then, the samples were located inside the oven with a temperature of 30°C for 23 hr. The changes in starch were recorded by a number ranging from 1 to 7. Then, the GC of the white rice was determined by transferring the rice into a pot of 0.2 normal potash, which indicated the movement of the gel of the cooked rice in millimeters (Vandeputte & Delcour, 2004). The PC of the rice flour was determined by the Kjeldahl procedure with a nitrogen conversion factor of 5.95 (Xu, Xiong, Li, & Zhao, 2008). To measure the physicochemical properties of the grain starch, samples were milled using a UDY cyclone miller (USA, Colorado) at 100 mesh. Then, three grams of each sample was separated. After that, 25 ml of distilled water was added to the separated samples. The resultant mixtures were then placed inside the metal cylinder of a rapid visco‐analyzer (RVA‐3D, Australia) (Kesarvani, Chiang, & Chen, 2016). Finally, the result of the test is a curve which shows the changes in the viscosity of the samples at various temperatures of cooking.

### 2.4 Experimental design and statistical analysis

The experiments were conducted in a historical design with a quadratic model, using RSM in Design‐Expert Software (Version 11), and the data were analyzed by ANOVA. The significance of the relationships between variables was determined via the analysis of variance, and the models were verified by various techniques including explanatory factors of *R* ^2^, adjusted *R* ^2^, and predicted *R* ^2^.

The responses could then be related to the factors by linear or quadratic models, enabling optimization. A quadratic model is shown in the following (Yousaf et al., 2017):

$$y=\beta_{0}+\sum_{i=1}^{k}\beta_{0}x_{i}+\sum_{i=1}^{k}\beta_{\mathrm{ii}}x_{i}^{2}+\sum_{i=1}^{k}\sum_{i=1}^{k}\beta_{\mathrm{ij}}x_{i}x_{j}$$

where *β*_0_, *β_i_*, and *β_ij_* are regression coefficients for intercept, linear, quadratic, and interaction coefficients, respectively, and *x_i_* and *x_j_* are coded independent variables.

Coefficient of variation (CV%), the standard deviation expressed as a percentage of the mean, calculated by dividing the Std. Dev by the mean and multiplying by 100. *SD*: (Root *MSE*) square root of the residual mean square. Consider this to be an estimate of the standard deviation associated with the experiment. Adequate precision: This is a signal‐to‐noise ratio. It compares the range of the predicted values at the design points to the average prediction error. Ratios greater than 4 indicate adequate model discrimination.

To optimize the quality properties of rice kernels, the independent variables were optimized using Design Express software (Version 11). In this study, the conditions of optimization for dependent and independent factors include the following: The highest value was selected for the response variables of PC (Allahgholipour, Ali, Alinia, Nagamine, & Kojima, 2006), PT (Mestres, Ribeyre, Pons, Fallet, & Matencio, 2011), Min.V (Kesarvani et al., 2016), and BD.V (Asante et al., 2013), and the lowest value was selected for GC (Kaur, Panesar, Bera, & Kumari, 2014), Pa.T (Kong et al., 2015), AC (Allahgholipour et al., 2006), SB.V (Asante et al., 2013), and F.V (Champagne, Bett‐Garber, Thomson, & Fitzgerald, 2009). Also, the conditions of optimization to predict the best quality properties of rice were selected the lowest values of the independent variables of F_0.05_, F_max_, D_max_, Et_0.05_, Et_max_, Es_0.05_, Es_max_, and E.F.

## 3 RESULTS AND DISCUSSIONS

### 3.1 Experimental design and model development

The analysis of variance (ANOVA) demonstrated that all of the independent variables affect the response variables (*p* < 0.01) and (*p* < 0.05) significantly. The regression equations for the response variables and P value, Std. Dev, coefficient of variation, adequate precision, and $R_{\text{pred}}^{2}$ values are presented in Table 1. In this study, only 17 response variables were predicted by the independent variables predicted *R* ^2^ ≥ 0.78. Koocheki, Taherian, Razavi, and Bostan (2009) suggested that for a well‐fitted model, *R* ^2^ should not be <0.80, while Chauhan and Gupta (2004) reported *R* ^2^ >0.75 as acceptable for fitting a model. Koocheki et al. (2009) claim that it is more appropriate to use adj *R* ^2^ of over 0.90 to assess the model accuracy. The model developed in this study indicated an adj *R* ^2^ level of higher than 0.90 (adj *R* ^2^ ≥ 0.91). In this study, adequate precision of all models was more than 4 and its CV% less. Effect of the independent variables on the regression equations are shown in Table 2. For example, in the response variable of AC, the independent variables D_max_ and Es_max_ have a more positive effect and the variable F_0.05_ has a negative effect on the regression equations, while the other independent variables have the lowest effect. All the presented equations in Table 1 are linear.

**Table 1:** Response surface equations in compressive tests

Treatment	Equations	*p*‐Value	$R_{\text{pred}}^{2}$	SD	CV%	A.P
*Domsiah* 9%	YPC = 11.67–37.9*A + 3.72*B + 1.15*D−084*E + 0.99*F−10.04*H	.004	0.93	0.01	0.12	19.61
*Dorfak* 9%	YMin.V = 243.9–64.3*A−50.3*B−477.35*C + 43.97*D−97.94*E + 131.75*G + 208.6*H	.02	0.95	0.03	0.02	102.8
	YF.V = 370.73–142.32*A−330.63*C + 6.1*D−8.8*E + 9.26*G + 9.83*H	.001	0.953	0.11	0.04	29.1
	YSB.V = 180.6–108.96*A + 6.43*B−245.1*C + 4.2*E + 7.9*D−7.9*G−9.53*H	.012	0.94	0.01	0.01	185.6
*Dorfak* 14%	YPC = 3.62 + 4.13*B−1.8*D + 3.6*E + 2.6*F−4.7*G−22.9*H	.012	0.82	0.04	0.59	12.2
	YBD.V = 31.6 + 279.7*A + 115.9*B−33.63*D + 82.53*E + 46.7*F−122.2*G−477.73*H	.004	0.94	0.16	0.17	38.4
*Hashemi* 14%	YP.T = 7.9–0.6*B−12.4*C−0.2*E−0.13*F + 0.4*G + 1.92*H	.005	0.86	0.01	0.1	17.41
*Kadus* 9%	YGC=−37.83 + 2.4*B + 313.3*C + 3.22*E + 2.15*F−5.83*G	.002	0.81	0.12	0.25	16.95
	YPC = 1.04 + 3.06*B + 18.06*C + 1.08*E + 0.66*F−2.17*G−9.4*H	.0011	0.91	0.01	0.13	31.1
	YMin.V = 98.94 + 13.9*B + 30.47*D−49.59*E−38.83*F + 54.92*G	.0014	0.78	0.3	0.23	15.98
	YSB.V = 115.1 + 1.98*D−1.15*E−1.13*F + 13.34*H	.0012	0.81	0.18	0.15	14.1
	YPa.T = 71.47–103.4*A + 20.6*B + 7*D−5.07*E−51.72*H	.0007	0.91	0.14	0.15	8.99
*Kadus* 14%	YAC = 41.54–146.6*A + 133.2*C + 1.8*D−9.75*E + 16.35*G−68.93*H	.0055	0.86	0.1	0.42	16.68
	YGC = 78.3 + 94.04*A + 2.42*D−5.42*E−8.36*F + 6.72*G	.0058	0.85	0.15	0.31	12.84
	YPC = 9.5–16.22*A−1.998*B + 20.34*C + 1.04*D−2.99*E−1.4*F + 4.5*G	.0052	0.86	0.01	0.1	38.02
	YMin.V = 185.83–397.5*A + 331.84*C + 6.8*D−27.01*E + 42.37*G−146.21*H	.0008	0.95	0.15	0.11	31.02
	YSB.V = 138.84–203.31*A + 183.54*C−7.05*E + 4.86*F + 13.49*G−73.7*H	.0053	0.92	0.12	0.1	18.8

Note

A.P = adequate precision, F_0.05_ = A, F_max_ = B, D_max_ = C, Et_0.05_ = D, Et_max_ = E, Es_0.05_ = F, Es_max_ = G, E.F = H.

**Table 2:** Effect of the independent variables on the regression equations

Response variable	Positive independent variables	Negative independent variables
AC (*Kadus* 14%)	D_max_, Es_max_	F_0.05_
GC (*Kadus* 14%)	F_0.05_, Es_max_	Es_0.05_, Et_max_
PC (*Domsiah* 9%)	F_max_	F_0.05_, EF
Min.V (*Dorfak* 9%)	Et_0.05_, Es_max_, EF	F_0.05_, F_max_, D_max_, Et_max_
FV (*Dorfak* 9%)	Et_0.05_, Es_max_, EF	F_0.05_, D_max_, Et_max_
SBV (*Dorfak* 9%)	F_max_, Et_0.05_	F_0.05_, D_max_, Es_max_, EF
BDV (*Dorfak* 14%)	F_0.05_, F_max_, Et_max_, Es_0.05_	Et_0.05_, Es_max_, EF
P.T (*Hashemi* 14%)	EF	D_max_
Pa. T (*Kadus* 9%)	F_max_	F_0.05_, EF

### 3.2 The effect of independent variables on response variables

**Figure 1:**
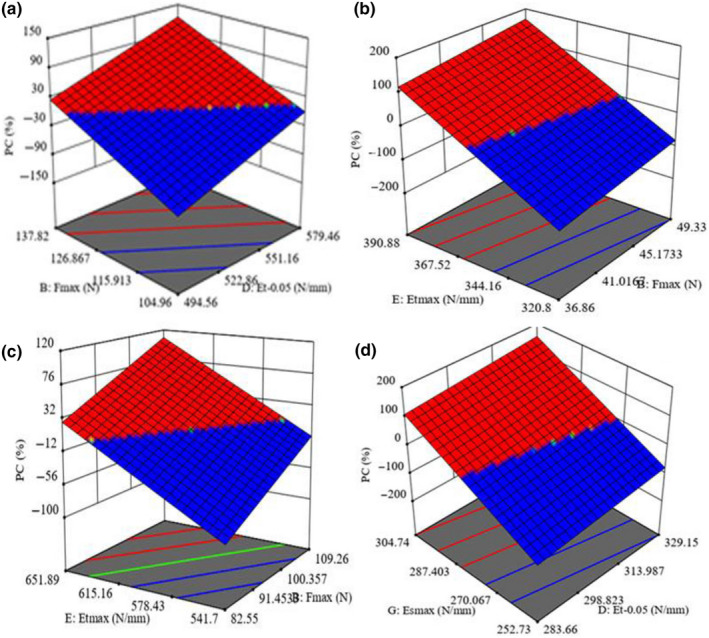
Statistical interaction effects of the independent variables on possibility of predicting the PC: (a) Fmax and F_0.05_, (b) F_max_ and Et_max_, (c) F_max_ and Et_max_, and (d) F_max_ and Et_max _on PC

**Figure 2:**
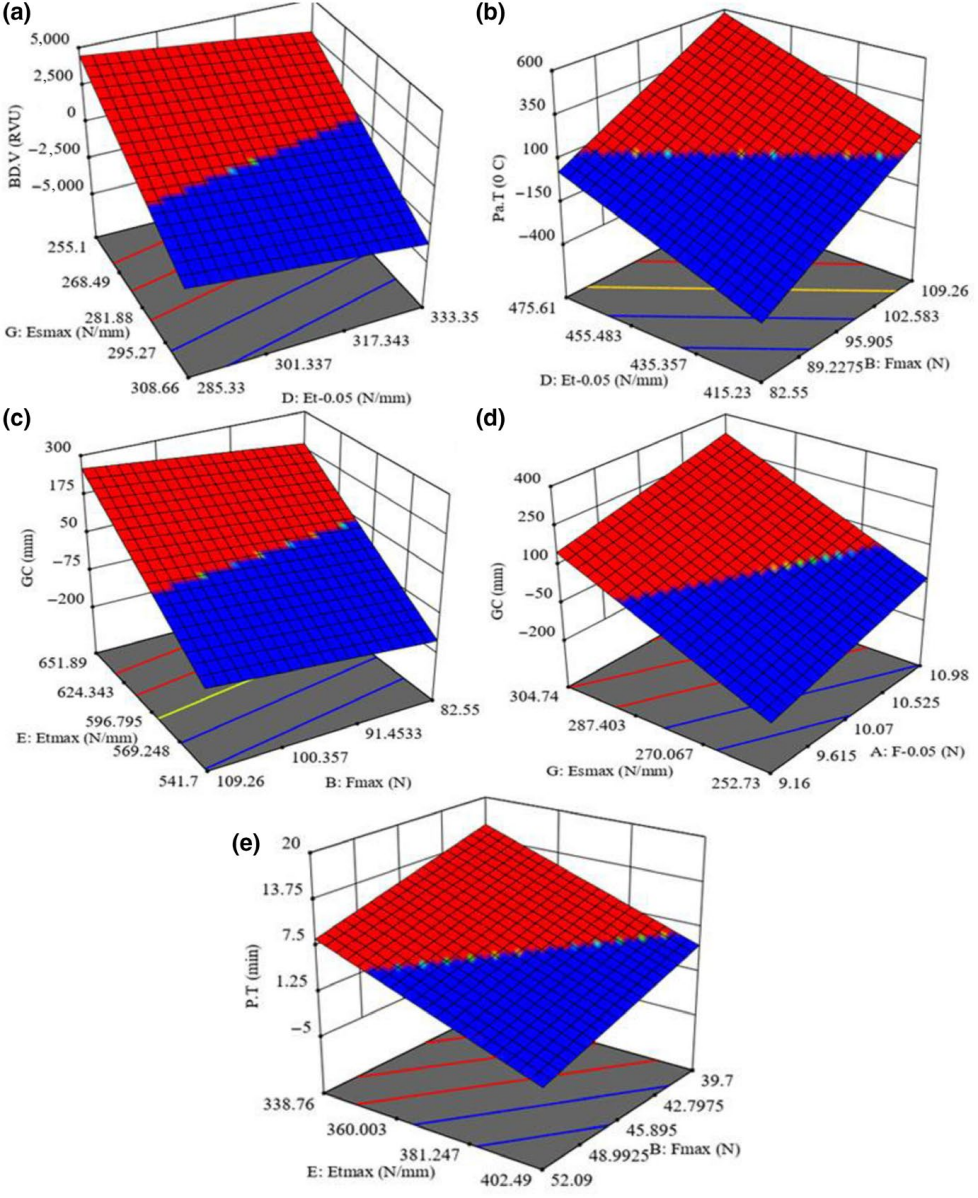
Statistical interaction effects of the independent variables on the possibility of predicting the response variables: (a) Et_max_ and Et_0.05_ on BD.V, (b) F_max_ and Et_0.05_ on Pa.T, (c) Et_max_ and F_max_, (d) F_0.05_ and Et_max _on GC, and (e) F_max_ and Et_max_ on PT

**Figure 3:**
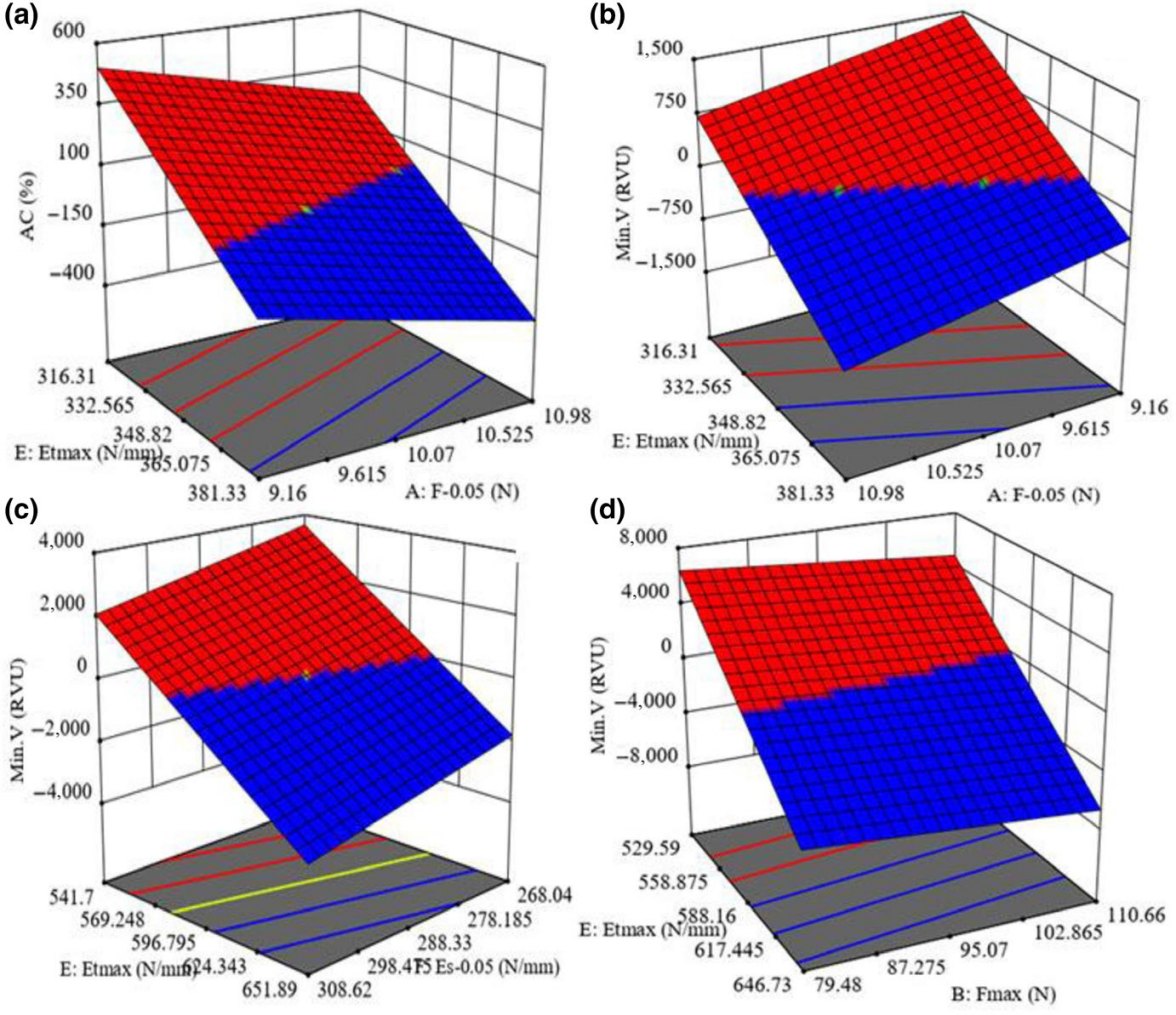
Statistical interaction effects of the independent variables on the possibility of predicting the response variables: (a) F_0.05_ and Et_max_ on AC, (b) F_max_ and Et_max_, (c) Et_max_ and Es_0.05_, and (d) Et_max_ and F_max_ on Min.V

**Figure 4:**
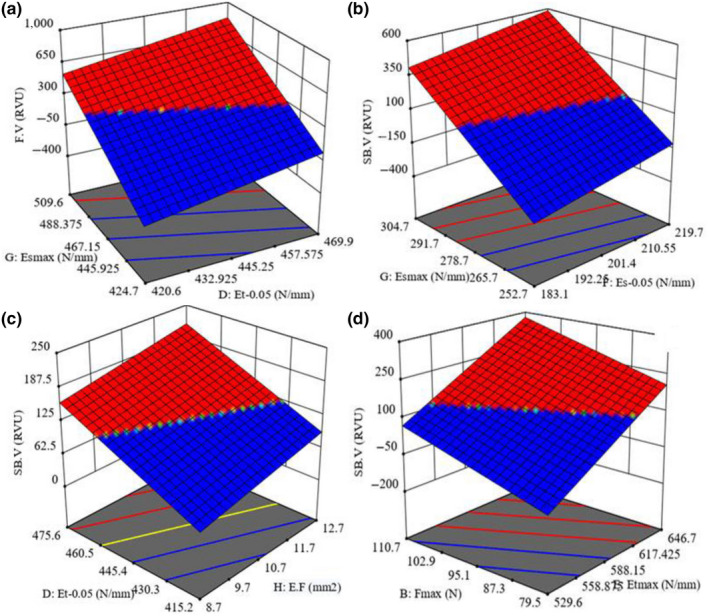
Statistical interaction effects of the independent variables on the possibility of predicting the response variables: (a) Es_max_ and Et_0.05_ on F.V, (b) Es_max_ and Es_0.05_, (c) EF and Et_0.05_, and (d) F_max_ and Et_max_ on SB.V

Statistical interaction effects of the independent variables on the possibility of predicting the response variables are shown in Figures 1, 2, 3, 4. In Figure 1a (*Domsiah* with MC of 9%), PC increased from 9.8% to 10% with the increase of F*_max _*from 104.96 to 137.82 N and Et_0.05_ from 494.56 to 579.46 N/mm. In Figure 1b (*Dorfak*, MC 14%), P.C increased from 6.4% to 6.82% with the increase of F_max_ from 36.9 to 49.3 N and Et_max_ from 320.9 to 390.9 N/mm. Similarly, in Figure 1c (*Kadus*, MC 9%), P.C increased from 8.1% to 8.37% with the increase of F_max_ from 82.6 to 109.3 N and Et_max_ from 541.7 to 651.9 N/mm. Finally, in Figure 1d (*Kadus*, MC 14%), P.C increased from 8.1% to 8.37% with an increase of Et_0.05_ from 283.7 to 329.2 N/mm and Es_max_ from 252.7 to 304.7 N/mm.

The PC of *Dorfak, Kadus*, and *Domsiah* were obtained to be 6.6%, 8.2%, and 9.9%, respectively. The rice grain mainly comprises carbohydrates, most of which are starchy and stored in the endosperm. After starch, PC is the second most important chemical compound in rice. High PC increases grain strength and transparency (Ogawa, 2008).

As the results indicate, the grain strength of *Dorfak* variety is lower as compared to *Kadus* and *Domsiah* cultivars. That is why the slopes of increase observed in F_max_ and Et_max_ of *Domsiah* and *Kadus* cultivars at the MC of 9% are higher than those of *Dorfak* variety. These findings are consistent with the results reported by Yan and Zhu (2001). As the MC increases from 12% to 18%, the strength properties of the rice grain decrease (Wouters & Baerdemaeker, 1988), which was confirmed by data observed in other researches (Cnossen, Jienez, & Siebenmorgen, 2003; Saiedirad et al., 2008). As a result, as Figure 1d shows, the slopes of variation in the variables related to *Kadus* variety at the MC of 14% are lower than those at the MC of 9%.

In Figure 2a (*Dorfak*, MC 14%), the BD.V value increased from 89.25 to 94.5 RVU with the decrease of Es_max_ from 308.7 to 255.1 N/mm and Et_0.05_ from 333.4 to 285.3 N/mm. Also, in Figure 2b (*Kadus*, MC 9%), Pa.T value increased from 92.45 to 94.5°C with the increase of F_max_ from 82.6 to 109.3 N and Et_0.05_ from 415.2 to 475.6 N/mm. In Figure 2c (*Kadus*, MC 9%), GC value increased from 48 to 49.5 mm with the increase of Et_max_ from 541.7 to 651.9 N/mm and Fmax from 82.6 to 109.3 N. Also, in Figure 2d (*Kadus*, MC 14%), GC value increased from 48 to 49.5 mm with the increase of Es_max_ from 252.7 to 304.7 N/mm and F_0.05_ from 9.26 to 10.98 N. In Figure 2e (*Hashemi*, MC 14%), PT value increased from 6 to 6.09 min with the decrease of F_max_ from 52.1 to 39.7 N and Et_max _from 402.5 to 338.8 N/mm.

By cooling down the samples by reducing the temperature, the soluble AC increases. As a result, the Min.V decreases to its lowest level and the BD.V increases (Allahgholipour et al., 2006). These findings are all consistent with the results reported by Kong et al. (2015). Consequently, the grains become soft and suitable leading to the decrease in their strength (Allahgholipour et al., 2006), which was confirmed by data observed in other researches (Asante et al., 2013). In this study, the mean BD.V in *Dorfak* variety was higher than other varieties except for *Kadus*. Since the mean PC in *Dorfak* was obtained 6.6, which is relatively lower, this finding is in line with the one reported by Champagne et al. (2009). Similarly, the mean Pa.T (93.56) in *Kadus* variety was lower than that of other varieties except for Dorfak. Moreover, its GT was higher than that of other varieties. On the other hand, Pa.T is negatively correlated with GT (Allahgholipour et al., 2006; Mestres et al., 2011). Similar results can be drawn from the research of Kong et al. (2015). Also, higher AC results in higher Pa.T (Sodhi & Singh, 2003) and more hardness (Allahgholipour et al., 2006; Kang, Kim, Kim, & Murata, 1994). As a result, the rupture force and the grains hardness decrease as the Pa.T decreases.

In Figure 2c,d, the GT in *Kadus* variety was estimated to be 3.52, which is considered relatively high. As a result, the grain strength is lower at the time of cooking (Mestres et al., 2011). Also, GC is negatively correlated with GT (Vandeputte & Delcour, 2004; Wang et al., 2010). Therefore, the grain strength at low GT and high GC is higher. Considering the AC, our results are similar to those reported by Kaur et al. (2014).

In Figure 2e, PT is suggestive of the time required for cooking the sample and reaching the PV. Pa.T has a reverse association with the GT, the higher the Pa.T, the lower the GT and the shorter the time required for cooking the sample (Fitzgerald, McCouch, & Hall, 2009; Mestres et al., 2011). These findings are consistent with the results reported by Kong et al. (2015). The obtained GT of the *Hashemi* variety was 4.5 which is considered to be in the intermediate to low range. The lower the GT, the shorter the cooking time (Fitzgerald et al., 2009). Mestres et al. (2011) showed that firmness decreases with an increase in the cooking time and firmness increases with a decrease in the GT. Therefore, the strength of the grain decreases when the increase in cooking time. These findings are in line with the findings reported by Loisel, Maache‐Rezzoug, Esneault, and Doublier (2006).

In Figure 3a (*Kadus*, MC 14%), AC increased from 23.52% to 24.86% with the decrease of Et_max_ from 381.3 to 316.3 N/mm and F_0.05_ from 10.98 to 9.2 N. In Figure 3b (*Kadus*, MC 14%), Min.V value increased from 131.3 to 134.9 RVU with the decrease of F_0.05_ from 10.98 to 9.2 N and Et_max_ from 381.3 to 316.3 N/mm. In Figure 3c (*Kadus*, MC 9%), Min.V value increased from 131.3 to 134.9 RVU with the decrease of Es*_0.05_* from 308.6 to 268.04 N/mm and Et_max_ from 651.9 to 541.7N/mm. Similarly, in Figure 3d (*Dorfak*, MC 9%), Min.V value increased from 162.9% to 164.9% with the decrease of F_max_ from 110.7 to 79.5 N and Et_max_ from 647.7 to 529.6 N/mm.

In Figure 3, rice with high AC can be either soft or firm after cooking, depending on its GC (Champagne et al., 1999), which was confirmed by data observed in other researches (Wang & Wang, 2001). The AC is negatively correlated with Min. V (Kesarvani et al., 2016), and high AC causes grain volume expansion (Ogawa, 2008). The increase of moisture in the cells close to the endosperm surface causes the cells to expand and compress together (Ogawa, 2008). Similar results can be drawn from the research of Cnossen and Siebenmorgen (2000). As a result, internal stress occurs, which causes internal failure in the grain; reduces the grain resistance (Cnossen & Siebenmorgen, 2000), which was confirmed by data observed in other researches (Cnossen et al., 2003); and consequently decreases F_max_ and Et_max_ values. The AC in *Dorfak* and *Kadus* varieties were obtained 23.74 and 24.25, respectively, which were higher than that of the other cultivars. Therefore, it is concluded that Min.V decreases by the increase of F_max_. It was also observed that as the MC increases from 9% to 14%, the slope of Fmax also increases. These findings are consistent with Mesters et al. (2011) finding that hardness decreases with the increase of viscosity.

In Figure 4a (*Dorfak*, MC 9%), the F.V value increased from 304.1 to 306.7 RVU with the increase of Es_max_ from 424.7 to 509.6 N/mm and Et_0.05_ from 420.6 to 469.9 N/mm.

In Figure 4b (*Kadus*, MC 14%), SB. V value increased from 125.4 to 127.3 RVU with the increase of Es_max_ from 252.7 to 304.7 N/mm and Es_0.05_ from 183.1 to 219.7 N/mm. Also, in Figure 4c (*Kadus*, MC 9%), SB.V value increased from 125.4 to 127.3 RVU with the increase of E.F from 8.7 to 12.7 J/mm^2^ and Et_0.05_ from 415.2 to 475.6 N/mm. As so, in Figure 4d (*Dorfak*, MC 9%), SB.V value increased from 125.4 to 127.6 RVU with the increase of Et_max_ from 529.6 to 646.7 N/mm and F_max_ from 79.5 to 110.7 N.

In Figure 4a, also, the mean values of AC and F.V were obtained to be 23.74% and 305.5 RVU, respectively. FV is positively correlated with AC (Allahgholipour et al., 2006). Cooked grains from the rice types with high amylose are usually firm and dry (Mestres et al., 2011; Wang et al., 2010). In this research, the AC of *Dorfak* was higher than that of all other cultivars. Therefore, the FV of this variety was higher and the grains were steady after cooking, and as a result, the grain strength was higher. Considering the PC, the results obtained in our study are similar to those reported by Champagne et al. (2009).

In Figure 4b‐d, different varieties have different levels of strength against stresses applied (Cnossen et al., 2003), which was confirmed by data observed in other researches (Cnossen & Siebenmorgen, 2000). In general, the SB.V in the modified varieties (*Dorfak* and *Kadus*) is higher than that of the local varieties (*Hashemi* and *Domsiah*). Moreover, the modified varieties have lower cooking quality and their grains become hard and stiff after cooking (Allahgholipour et al., 2006). In this study, the mean SB.V of *Dorfak* and* Kadus* varieties was higher than that of all other varieties. In Figure 4c, the E.F is positively correlated with F_max _and the grain strength (Li et al., 2014). Therefore, E.F changes with the changes in F_max_. On the other hand, it has been observed that cultivars showing low‐soluble AC generally have higher SB.V, lower BD.V, harder GC (Allahgholipour et al., 2006; Kaur et al., 2014), and harder texture after cooking (Allahgholipour et al., 2006), which was confirmed by data observed in other researches (Sodhi & Singh, 2003).

### 3.3 Optimization

Input means (IM) and results of optimal values (OV) of response and independent variables in the compressive tests are shown in Table 3. Ten solutions were developed to determine the optimal conditions for the data extracted from the compression test. The software determined the optimal conditions of the independent variables based on the maximum goal and minimization of the response variables. Using the desirability function method, the optimum extrusion conditions were obtained. The desirability value was obtained more than 0.80. The optimal values of the independent variables of F_0.05_, F_max_, D_max_, Et_0.05_, Et_max_, Es_0.05_, Es_max_, and EF to predicting of the response variable of PC (Domsiah with MC 9%) was as 16.49, 105.0, 0.23, 498.5, 654.8, 328.86, 579.9, and 11.52, respectively. As shown in Table 3, the optimal values of the independent variables have a decreasing trend, and the optimal values of the response variables are proportional to the optimal conditions. Also, the advantage of optimization conditions is in Table 3 the optimal use of independent variables in predicting response variables.

**Table 3:** Input mean (IM) and results of optimal values (OV) of response and independent variables in the compressive tests

Response variables	Optimal conditions of independent variables								Optimal conditions of response	Desirability
	F_0.05_	F_max_	D_max_	Et_0.05_	Et_max_	Es_0.05_	Es_max_	E.F		
*Domsiah* 9%(PC)										
IM	17.8	123.6	0.23	537.2	728.5	356.1	581.0	14.36	9.91	
OV	16.49	105.0	0.23	498.5	654.8	328.86	575.9	11.52	10	0.95
*Dorfak*9% (Min.V)										
IM	14.78	98.4	0.23	450.9	603.6	295.5	479.5	11.1	163.77	
OV	14.11	85.4	0.2	440.0	549.6	291.8	439.2	8.86	164.95	0.80
*Dorfak* 9% (F.V)										
IM	14.78	98.4	0.23	450.9	603.6	295.5	479.5	11.1	305.47	
OV	14.42	95.07	0.21	420.6	536.4	295.51	435.95	8.1	304.12	0.89
*Dorfak* 9% (SB.V)										
IM	14.78	98.4	0.23	450.9	603.6	295.5	479.5	11.1	126.49	
OV	14.74	79.48	0.21	420.6	547.7	289.53	442.23	8.14	162.66	0.83
*Dorfak* 14% (PC)										
IM	10.29	43.56	0.2	312.1	359.5	205.9	285.7	3.64	6.6	
OV	10.36	36.9	0.17	286.5	320.8	195.9	256.51	3.29	6.82	0.91
*Dorfak* 14% (BDV)										
IM	10.29	43.56	0.2	312.1	359.5	205.9	285.7	3.64	92.15	
OV	9.46	38.42	0.17	310.0	329.6	195.14	258.03	2.91	64.52	0.84
*Hashemi* 14% (PT)										
IM	10.19	44.68	0.17	312.6	364.4	203.8	288.2	3.80	6.04	
OV	9.99	42.02	0.16	313.2	347.8	179.5	269.02	3.56	6.09	0.89
*Kadus* 9% (GC)										
IM	14.3	94.32	0.22	439.3	586.5	285.9	464.8	10.5	48.86	
OV	14.41	84.6	0.21	445.4	541.8	268.04	429.14	9.4	48	0.98
*Kadus* 9% (PC)										
IM	14.3	94.32	0.22	439.3	586.5	285.9	464.8	10.5	8.22	
OV	14.41	82.6	0.22	445.4	541.7	279.6	430.3	9.03	8.37	0.88
*Kadus* 9% (Min.V)										
IM	14.3	94.32	0.22	439.3	586.5	285.9	464.8	10.5	133.35	
OV	14.41	82.55	0.22	415.2	556.9	271.3	444.1	8.86	134.95	0.92
*Kadus* 9% (SB.V)										
IM	14.3	94.32	0.22	439.3	586.5	285.9	464.8	10.5	126.51	
OV	14.41	95.9	0.22	416.1	541.7	271.42	471.9	8.75	125.35	0.96
*Kadus* 9% (Pa.T)										
IM	14.3	94.32	0.22	439.3	586.5	285.9	464.8	10.5	93.56	
OV	13.4	83.69	0.22	416.3	543.7	288.3	471.9	9.17	92.45	0.96
*Kadus* 14% (AC)										
IM	9.85	40.61	0.16	299.8	342.8	197.1	271.8	3.33	24.25	
OV	9.75	41.7	0.15	286.2	316.3	201.4	254.6	2.82	23.52	0.91
*Kadus* 14% (GC)										
IM	9.85	40.61	0.16	299.8	342.8	197.1	271.8	3.33	48.86	
OV	9.16	41.7	0.16	283.7	319.8	184.3	252.7	3.94	48	0.95
*Kadus* 14% (PC)										
IM	9.85	40.61	0.16	299.8	342.8	197.1	271.8	3.33	8.22	
OV	9.16	35.95	0.15	283.7	352.7	183.14	273.3	3.17	8.37	0.81
*Kadus* 4% (Min.V)										
IM	9.85	40.61	0.16	299.8	342.8	197.1	271.8	3.33	133.35	
OV	9.16	41.7	0.16	290.5	323.0	201.4	253.2	2.99	134.95	0.86
*Kadus* 14% (SB.V)										
IM	9.85	40.61	0.16	299.8	342.8	197.1	271.8	3.33	126.51	
OV	9.16	41.7	0.15	306.4	327.3	186.8	254.1	2.78	125.35	0.94

Note

Units: F_0.05_ and F_max_(*N*), D_max_(mm), Et_0.05_, Et_max_, Es_0.05_ and Es_max_(*N*/mm) and E.F(J/mm2), Quality properties units: AC(%), GT(none), GC(mm), PC(%), Min.V, P.V, F.V, BD.V and SB.V (RVU: rapid visco‐analyzer units), PT(min), Pa.T(°C).

Abbreviations: IM = input mean, OV = optimal values.

## 4 CONCLUSION

The present study investigated the prediction of some quality properties of white rice via the use of a compression test. It examined four varieties of white rice, namely *Domsiah, Hashemi, Dorfak*, and *Kadus* in two moisture levels. The results showed that RSM is one of the most useful methods in predicting the quality properties of rice based on the strength properties of a single kernel. According to the findings, it is possible to perform some simple tests on the strength of a single kernel using F_max_, E.F, F_0.05_, Et_max_, Et_0.05_, Es_max_, and Es_0.05_ and predict some of the most important quality components of rice in bulk, such as the AC, GT, GC, PC, Min.V, PV, FV, BD.V, SB.V, PT, and Pa.T, which are all considered as important quality indicators in the marketing of this product. The most appropriate model for response variables prediction of AC (_Kadus_ with MC 14%), GC (*Kadus* MC 14%), PC (_Domsiah_ MC 9%), Min.V, FV, and SBV (_Dorfak_ MC 9%), BDV (_Dorfak_ MC 14%), PT (_Hashemi_ MC 14%), and Pa.T (_Kadus _MC 9%) was *R* ^2^ (0.98, 0.96, 0.98, 0.99, 0.99, 0.99, 0.99, 0.98, and 0.98), and adequate precision as 16.68, 12.84, 19.61, 102.8, 29.1, 185.6, 38.4, 17.41, and 8.99, respectively, with the most appropriate optimization conditions (23.52%, 48, 10%, 164.95 RVU, 304.12 RVU, 162.66 RVU, 64.52 RVU, 6.09 min, and 92.45°C) with desirability more than 0.80. Also, the results showed that the optimal values of the independent and response variables are proportional to the optimal conditions. This finding will pave the way for designing and developing devices to test the properties of single kernels of rice, similar to those that are now being employed to examine the kernels of other products such as wheat.

## CONFLICT OF INTEREST

None declared.
